# Preventive Effects of Neuroprotective Agents in a Neonatal Rat of Photothrombotic Stroke Model

**DOI:** 10.3390/ijms21103703

**Published:** 2020-05-24

**Authors:** Yoon Young Yi, Hyo Jung Shin, Seung Gyu Choi, Joon Won Kang, Hee-Jung Song, Sung Koo Kim, Dong Woon Kim

**Affiliations:** 1 Department of Pediatrics, Hallym University and Kangdong Sacred Heart Hospital, Seoul 05355, Korea; yooroung82@naver.com; 2 Department of Medical Science, Chungnam National University, Daejeon 35015, Korea; shinhyo1013@gmail.com (H.J.S.); csk950802@gmail.com (S.G.C.); childlove@cnu.ac.kr (J.W.K.); nrsono@cnuh.co.kr (H.-J.S.); 3 Department of Anatomy and Cell Biology, Chungnam National University, Daejeon 35015, Korea; 4 Brain Research Institute, Chungnam National University, Daejeon 35015, Korea; 5 Department of Pediatrics, Chungnam National University Hospital, Daejeon 35015, Korea; 6 Department of Neurology, Chungnam National University and Sejong Hospital, Sejong 30099, Korea; 7 Department of Pediatrics, Hallym University and Dongtan Sacred Heart Hospital, Hwasung Gyunggi-do 18450, Korea

**Keywords:** photothrombotic stroke, neonatal period, Aspirin, clopidogrel, Coenzyme Q10, neuroprotection

## Abstract

Neonatal ischemic stroke has a higher incidence than childhood stroke. Seizures are the first sign for the need for clinical assessment in neonates, but many questions remain regarding treatments and follow-up modalities. In the absence of a known pathophysiological mechanism, only supportive care is currently provided. Stroke-induced microglia activation and neuroinflammation are believed to play a central role in the pathological progression of neonatal ischemic stroke. We induced a photothrombotic infarction with Rose Bengal in neonatal rats to investigate the effects of pre- and post-treatment with Aspirin (ASA), Clopidogrel (Clop), and Coenzyme Q10 (CoQ10), which are known for their neuroprotective effects in adult stroke. Pre-stroke medication ameliorates cerebral ischemic injury and reduces infarct volume by reducing microglia activation, cellular reactive oxygen species (ROS) production, and cytokine release. Post-stroke administration of ASA, Clop, and CoQ10 increased motor function and reduced the volume of infarction, and the statistical evidence was stronger than that seen in the pre-stroke treatment. In this study, we demonstrated that ASA, Clop, and CoQ10 treatment before and after the stroke reduced the scope of stroke lesions and increased behavioral activity. It suggests that ASA, Clop, and CoQ10 medication could significantly have neuroprotective effects in the neonates who have suffered strokes.

## 1. Introduction

Pediatric ischemic stroke occurs more frequently in neonates with the incidence of 1 per 4000 live births, compared to childhood incidence of 1 per 100,000 children [1]. Perinatal ischemic stroke is usually defined as a focal cerebral arterial or venous occlusion, occurring between 20 weeks of gestational age and 28 postnatal days. Although severe disability in neonates with stroke is lower than that in adults, many children have mild to moderate motor impairment and long-term developmental problems. Thrombolytic therapy within 3 h of the onset of stroke symptoms is the most effective therapeutic approach for acute stroke management in over two-year-old patients [2], but not for neonates. This is due to complications and high mortality after treatment and relatively lower recurrence rates (1.2–2.8%) [3,4] than other age groups (20–30%) [5]. Nevertheless, neonates with high-risk factors such as congenital heart disease may still have recurrent stroke, and cardiac disorders are associated with 10% to 30% of strokes in children [6,7].

In an adult rat stroke model, conventional hypoxic-ischemic (HI)-based methods can be technically challenging due to uncontrollable infarct volume and high mortality rates, and the P7 rat brain is highly resistant to hypoxia [8]. Recently, there have been large scale studies on the early detection of collateral blood vessels using Doppler ultrasound imaging in neonatal rats, which may help reduce the variability [9]. However, there is still a need for some specific techniques and the need for a larger number of rats.

Maxwell and Dyck introduced a noninvasive and reproducible focal ischemic stroke model in mice with Rose Bengal (RB) and laser-induced photothrombosis [8]. RB is a photosensitive agent, inducing a coagulation cascade, which produces an ischemic lesion that is pathologically relevant to clinical stroke [10]. Although its main mechanism includes edema from endothelial damage, it may be a useful tool to investigate the effect of neuroprotective drugs in a small focal cortical area in the small brain without other compounding factors.

We investigated the effects of commonly used and possibly neuroprotective medication, acetylsalicylic acid (ASA; Aspirin), clopidogrel (Clop), and Coenzyme Q10 (CoQ10, ubiquinone, ubidecarenone) in RB-induced photothrombosis in neonatal rats. ASA and Clop have been the most widely used antiplatelet drugs for the prevention of ischemic stroke. ASA produces some benefit in long-term outcome and survival if administered within 14 days of stroke onset [11]. Although its effects come from antiplatelet actions through the cyclooxygenase (COX)-dependent pathway, directive neuroprotective effects have also been reported in several studies [12]. Clop is a commonly prescribed drug with ASA or alone in adolescence and adult stroke patients. It inhibits adenosine diphosphate (ADP)-P2Y12 receptors that may affect microglial cytotoxicity in the ischemic brain [13]. CoQ10 is an essential cofactor present in all mitochondria-containing cells in the respiratory chain, which transfers electrons from respiratory complex I and II to complex III, creating a transmembrane proton gradient and driving phosphorylation of adenosine diphosphate [14]. Belousova et al. reported the effectiveness of intravenous treatment with CoQ10 in a transient middle cerebral artery occlusion (tMCAo) rat model [15]. Based on the evidence above, we aimed to develop a focal cerebral ischemic stroke model in P7 rats and imply on pre- and post-stroke treatment with ASA, Clop, and CoQ10. To our knowledge, this is first demonstration of neuroprotective medication, with the commonly used drug, ASA, Clop, and CoQ10 in RB-induced photothrombosis in neonatal rats.

## 2. Results

### 2.1. Focal Ischemic Stroke in Neonatal Rat Induced by Photothrombosis

**Figure 1:**
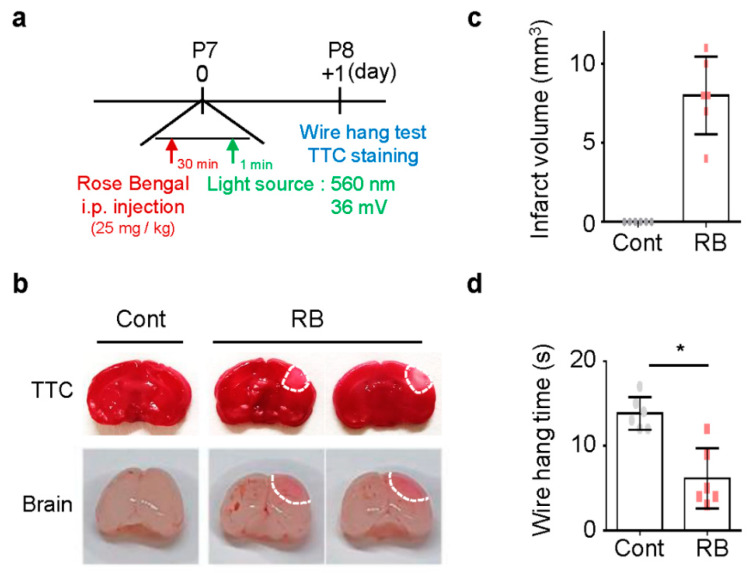
Damage following Rose Bengal (RB) photothrombosis in perinatal rat model. (**a**) Experimental design for animal model. Photothrombosis is achieved via intraperitoneal injection of RB (25 mg/kg) and illumination (532 nm, 36 mV) of the sensory-motor cortex through a cranial window. (**b**) Representative photographs of coronal brain sections stained with 2,3,5-TriphenylTertrazoium Chloride (TTC) and whole-brain at one day after modeling. The lack of formazan production (white tissue) indicates an infarct area with a white line. Quantification of (**c**) infarct volume and (**d**) wire hang test. Data represented as mean ± SD. * *p* < 0.01 compared with the control group.

To study the focal ischemic stroke of neonatal rats, RB was injected intraperitoneal injection (i.p.) in P7 rats, and blue laser light-induced photothrombosis in the RB group (Figure 1a) [8]. No pups died during the procedure. Tissue viability and morphologic changes of the photochemically induced stroke were evaluated by 2,3,5-TriphenylTertrazoium Chloride (TTC) staining [16]. TTC staining revealed a selective focal ischemic lesion in the right sensorimotor cortex area (Figure 1b). The TTC-negative region was significantly well-demonstrated on the first day after stroke induction (Figure 1c). The wire hanging time was shorter in the RB-injected group (*n* = 6, 6.17 ± 3.54) than in the control group (*n* = 6, 13.83 ± 1.94; *p* = 0.02) (Figure 1d).

**Figure 2:**
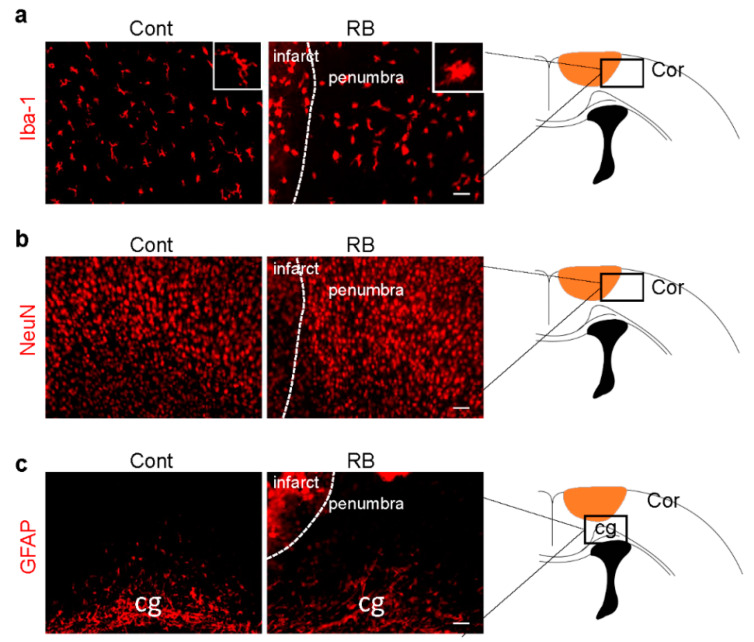
Microglia activation increased in the RB photothrombotic stroke but not in astrocytes and neurons. (**a**) Brain tissues were immunostained with anti-Iba-1 (microglial marker), (**b**) GFAP (astrocytic marker), and (**c**) NeuN (neuronal marker) antibodies. The white box shows microglia morphology under higher magnification. Scale bar = 100 µm. Abbreviations: Cont: control, Cor: cortex, cg: cingulated gyrus, Iba-1: isonized calcium-binding adaptor molecule 1, GFAP: glial fibrillary acidic protein, NeuN: neuronal nueclei

Microglia activation is an early response to brain ischemia [17]. Recent studies have revealed that microglial cells undergo morphological transformation after focal arterial stroke in the neonatal brain [18]. Another study showed that astrocytes took longer to reactivate than microglia [19]. After the brain was immunostained with a microglia marker, Iba-1, we found that the morphology of microglia changed from a ramified to an activated form in our stroke model (Figure 2a). There was no significant difference in astrocytes and neurons in the penumbra, suggesting microglia activation was the only cellular change one day after RB-induced neonatal stroke (Figure 2b,c).

### 2.2. Pre-Stroke Medication Ameliorates Cerebral Ischemic Injury and Reduces Infarct Volume by Reducing Cellular ROS Production and Cytokine Release

**Figure 3:**
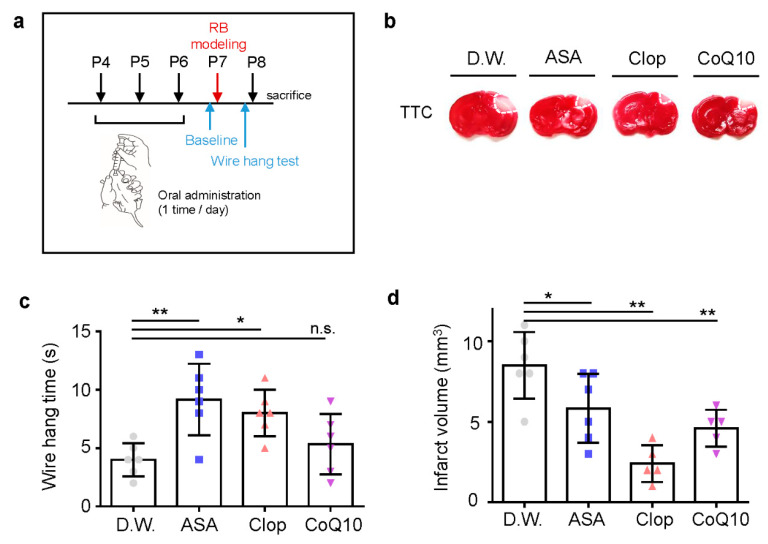
Neuroprotective effects of drug pre-treatment on infarct volume and behavioral function in a neonatal rat model of photothrombotic stroke. (**a**) Experimental design for drug treatment by oral administration and RB animal modeling. (**b**) Representative images of coronal brain sections stained with TTC. (**c**) Grip test for time assay, as assessed 24 h after RB modeling. (**d**) Brain infarct volumes in rats after 24 h are presented as mean ± SD. ** *p* < 0.005; * *p* < 0.01 compared with the D.W. group. Abbreviations: D.W.: distilled water, ASA: acetylsalicylic acid, Clop: clopidogrel, CoQ10: coenzyme Q10, n.s.: not significant.

To confirm the potent neuroprotective effects of drugs pre-treatment *in vivo*, we used oral administration of drugs in a photothrombotic ischemic mouse model. ASA, Clop, CoQ10, or autoclaved pure water was administered once a day before stroke modeling for three days (Figure 3a). Pre-treatment with all drugs resulted in significantly decreased infarct volume due to ischemic insult (Figure 3b). It also improved motor function in ASA (*n* = 6, 9.17 ± 3.06) and Clop (*n* = 6, 8.00 ± 2.00), not in CoQ10 (*n* = 6, 5.33 ± 2.58) compared to the distilled water (D.W.) group (*n* = 6, 4.00 ± 1.41; *F* = 6.140, *p* = 0.0039) (Figure 3c). Furthermore, pre-stroke administration of ASA (*n* = 6, 5.83 ± 2.14), Clop (*n* = 5, 2.4 ± 1.14), and CoQ10 (*n* = 5, 4.6 ± 1.14) reduced the infarction volume compared to the vehicle group (*n* = 6, 8.5 ± 2.07; F = 11.70, *p* = 0.0002) (Figure 3d).

**Figure 4:**
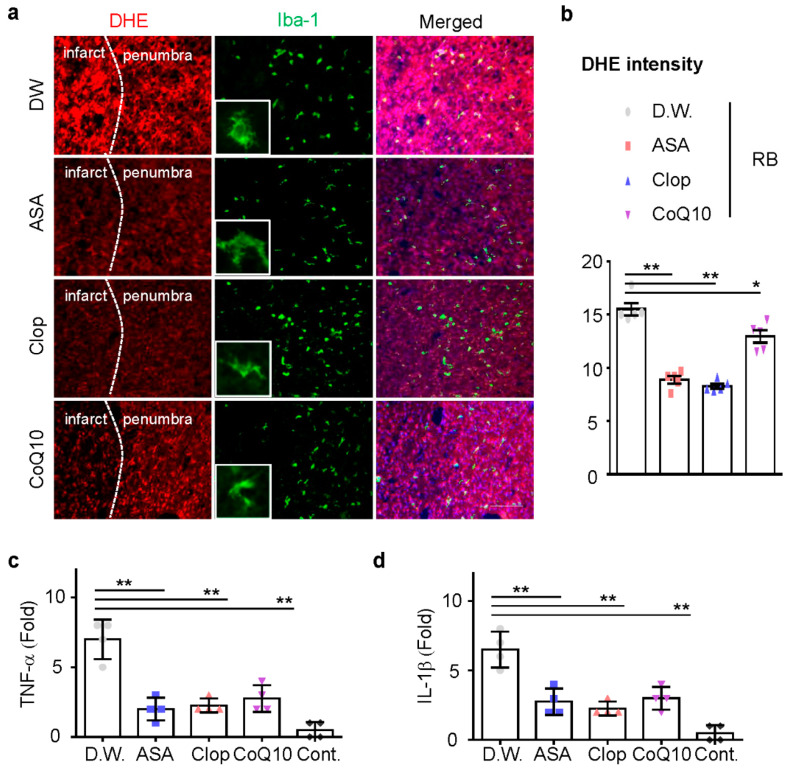
Reactive oxygen species (ROS) production and microglia activation in the penumbra. (**a**) Representative images of ROS-dependent Dihydroethidium (DHE) fluorescence in the penumbra part of the infarct brain. (*n* = 5, total *n* = 20) (**b**) DHE staining density measured using the Image J software. The total mRNA was isolated from the cortex of the ipsilateral brain at day 1 after RB modeling and utilized for cDNA synthesis. The expression of TNF-α (**c**) and IL-1β (**d**) mRNA was measured by qRT-PCR (*n* = 4, total *n* = 16). Data are presented as the mean ± SD. ** *p* < 0.005; * *p* < 0.01 compared with the D.W. group. Scale bar = 50 µm.

Next, we investigated whether ASA, Clop, and CoQ10 could reduce microglia activation and inflammation in this model. ASA, Clop, and CoQ10 significantly decreased the activity of microglia (Figure 4a). These results support the idea that brain injury caused by ischemic stroke could increase the production of infarct reactive oxygen species (ROS). Dihydroethidium (DHE) staining revealed that the administration of drugs attenuated ROS production in the penumbra (Figure 4b). Previous neonatal transient MCAO rodent models showed a rapid expression of interleukin (IL)-1β after HI injury [20,21]. We, therefore, investigated whether the reduction of microglia activation could downregulate mRNA expression of these genes, including TNF-α and IL-1β. mRNA expression of pro-inflammatory genes was tested in all drug-treated group and the expression of mRNA decreased after drug pretreatment (Figure 4c,d). These findings demonstrate that ischemic preconditioning tends to reduce ROS formation in neonatal ischemic rat brain.

### 2.3. Post-Stroke Medication Also Has Protective Effects on Neonatal Stroke

**Figure 5:**
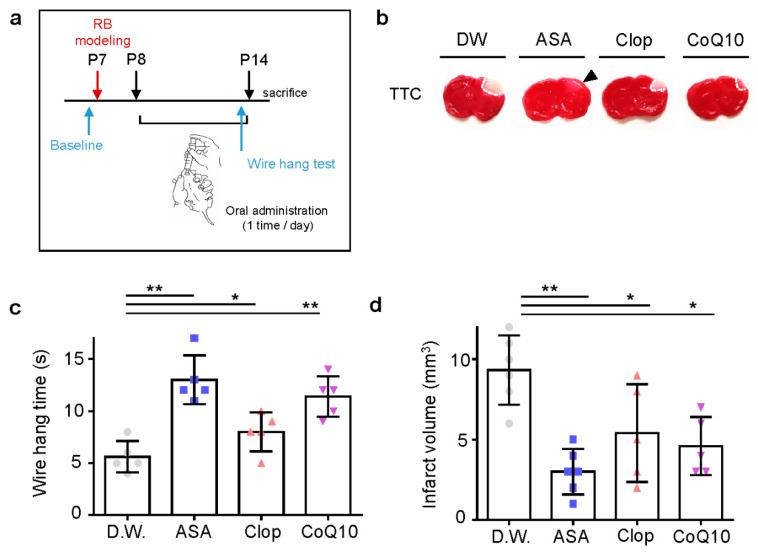
Neuroprotective effects of drugs post-treatment on the infarct volume and behavioral function in a neonatal rat model of photothrombotic stroke. (**a**) Experimental design for drugs by oral administration and RB animal modeling. (**b**) Representative images of coronal brain sections stained with TTC. (**c**) The length of time until the rat fell from the wire was recorded at perinatal day 14 after RB photothrombosis and after drug treatment for seven days. (**d**) Brain infarct volumes in rats after 24 h are presented as mean ± SD. ** *p* < 0.005; * *p* < 0.01 compared with the D.W. group.

As anti-thrombotic drug administration timing is crucial for its efficacy in adult stroke, we predicted this would be even more so in neonatal stroke. To determine the effects of medication post-stroke compared to pre-stroke, each drug was administered once a day for seven days after stroke modeling (Figure 5a). In all drug treatments, the infarct volume decreased significantly (Figure 5b). CoQ10 is an antioxidant, acting as a free radical scavenger and protecting cells from oxidative damage [22]. ASA appeared to have a greater effect on infarct volume when administered post-stroke compared to pre-stroke. Post-stroke administration of ASA, Clop, and CoQ10 (13 ± 2.35, 8 ± 1.87, and 11.4 ± 1.95; each *n* = 5) showed higher motor function than D.W. group (5.6 ± 1.52, *n* = 5; *F* = 14.71, *p* < 0.0001) (Figure 5c) and reduced the volume of infarction (3 ± 1.41, *n* = 6; 3.8 ± 1.30, *n* = 5; and 3.2 ± 0.84, *n* = 5 vs. 9.33 ± 2.16, *n* = 6; *F* = 22.36, *p* < 0.0001) (Figure 5d).

## 3. Discussion

Neonatal stroke has a lower recurrent rate than in adults, but recurrent events still exist and there is a need for treatment. Systemic thrombolysis using tissue plasminogen activator is an effective therapy for stroke but its time window is short and only for adolescents or adults. For this reason, it is important to have an effort to minimize damage after stroke with preventive medications. The effect of preventive medication for cerebral ischemia has been analyzed in adults but rarely at the immature brain stage.

Blood restore of the brain leads to severe damage to insulted neurons rather than protection. This contradictory phenomenon was called cerebral ischemia-reperfusion injury [23]. It has been established that its mechanisms are associated with oxidative stress, excitotoxicity, inflammation, and edema [23,24]. Among them, oxidative stress is the most common and well-investigated factor in cerebral ischemia-reperfusion injury. However, its exact mechanism is not fully known [25].

Neuroinflammation plays an important role in ischemic brain injury, and modulating the microglia-mediated inflammatory response to ischemia has been considered a potential target for neuroprotective intervention [26]. The post-ischemia inflammatory response is initiated by glial cell activation, peripheral leukocyte infiltration, and damage-associated molecules such as high-mobility group protein 1, nucleic acid fragments, nucleotides, and purines [27]. Microglia are responsible for the secretion of pro-inflammatory cytokines such as TNF-α, IL-1β, and IL-6, and they also induce the activation of inducible nitric oxide synthase (iNOS). This increases the production of ROS, compromising the integrity of the blood brain barrier (BBB), and promoting neuronal apoptosis [28,29]. Larger infarct size in these animals is associated with additional accumulation of several pro-inflammatory cytokines and chemokines [30]. Excessive ROS levels cause both functional and structural impairment of brain tissue and play a pivotal role in the pathogenesis of cerebral ischemia [31]. The signaling pathways by which ROS contributes to the pathophysiology of ischemic stroke in neonatal periods are complex and require further investigation.

In the immature brain, microglia proliferate and evolve to an amoeboid, activated phenotype a few hours after an ischemic insult, which correlates with the massive release of pro-inflammatory cytokines [32]. In a previous study, the P9 brains demonstrated a dramatic increase in microglia activation and a predominantly pro-inflammatory cytokine response early after HI, suggesting that inhibition of microglia may provide benefit in immature brains when administered soon after injury. In contrast, despite similar levels of apoptotic cell death early after injury, the P30 mice demonstrated less microglia activation and a more balanced pro- vs. anti-inflammatory response after HI, suggesting that inhibition of microglia in juvenile brains may be less effective or possibly worsen the outcome after HI [26]. The neonatal brain is prone to ROS after HI [33].

An age-appropriate rodent model is important for the prediction of biochemical and neuroanatomical changes during early ischemic events. Most of the previous studies, which showed inflammatory responses in neonatal rodents, used permanent middle cerebral artery (MCA) electrocoagulation with transient ipsilateral common carotid artery occlusion, or transient filament MCA occlusion stroke models [30,34,35,36,37]. The conventional MCAO model produces a large infarction volume, and usually showed high mortality. Additionally, variation in lesion size of this model tends to be large, ranging from 5% to 50% of the cerebral hemisphere [38]. Recent studies have shown controllable methods for the detection of collateral circulation in the conventional neonatal stroke model [9,39]. However, it has a large volume to explore penumbra area, which is the target of this study. Photothrombotic cerebral infarction has high reproducibility in a small size-brain and with exact location [40]. Therefore, we chose RB-induced photothrombosis as a method in the neonatal stroke rat model.

The basic mechanism of ASA and Clop in the prevention of stroke is antiplatelet effects, and CoQ10 is usually mentioned in primary mitochondrial disease or secondary deficiency state. However, ASA, Clop, and CoQ10 also have shown neuroprotective effects in several previous adult rat models. ASA upregulates proteins such as ceruloplasmin, which is an effective antioxidant in the CNS that protects neural cells from oxidative stress in vitro [41]. Clop’s active metabolite crosses the BBB at a level that can affect P2Y12-mediated microglial responses [42]. Clop antagonizes the platelet P2Y12 receptor, which is primarily expressed in microglia, but not in astrocytes or neurons. CoQ10 acts as a free radical scavenger and protects cells from oxidative damage [22]. Pretreatment with CoQ10 may protect neuronal cells against oxidative stress, stabilizing the mitochondrial membrane, and reducing the amount of mitochondrial ROS generation [43]. Oral administration of CoQ10 can inhibit cytochrome c release from mitochondria in mouse brain synaptosomes [44].

First, we focused on assessing whether pre-stroke exposure to ASA, Clop, and CoQ10 reduces infarct size in a neonatal stroke model. All of these three drugs showed reduced infarct volume and increased time of wire hanging test. The statistical significance of ASA was the strongest compared to the others in the wire hanging test, and showed the most statistical significance in reduced ROS production and inflammatory markers. However, ASA showed the least decreased infarct volume among the three drugs. Second, we compared pre- and post-stroke medication administration. Post-stroke exposure to ASA, Clop, and CoQ10 reduced the infarct size and increased the wire hanging time. Of the three drugs, ASA showed the most statistical significance when compared to the vehicle group. Overall, the neuroprotective agents used in our study were able to reduce the infarction volume and improve the result of the behavioral test. However, the decreased infarction volume was not proportional to the increase in hanging time.

Our study presented is the first to present a promising study to treat and prevent ischemic stroke in neonatal periods using drugs which are prescribed to adults. This study shows that ASA, Clop, and CoQ10 can significantly reduce infarct volume when administered immediately after photothrombosis compared to vehicle-treated rats. These considerations are beyond the scope of this study and will be investigated in future work from our research group.

We investigated whether ASA, Clop, and CoQ10 could reduce microglial activation and inflammation in this model. ASA, Clop, and CoQ10 significantly decreased the activity of microglia (Figure 4a). These results support the idea that brain injury caused by ischemic stroke could increase the production of infarct reactive oxygen species (ROS). DHE staining revealed that the administration of drugs attenuated ROS production in the penumbra (Figure 4b). Previous neonatal transient MCAO rodent models showed a rapid expression of interleukin (IL)-1β after HI injury [20,21]. We, therefore, investigated whether the reduction of microglia activation could downregulate mRNA expression of these genes, including TNF-α and IL-1β. mRNA expression of pro-inflammatory genes was tested in all drug-treated groups, and the expression of mRNA decreased after drug pretreatment (Figure 4c,d). These findings demonstrate that ischemic preconditioning tends to reduce ROS formation in the neonatal ischemic rat brain.

## 4. Materials and Methods

### 4.1. Animals

P7 Sprague-Dawley rats purchased from Damul Science Korea (Daejeon, Korea) were housed at 23 °C under controlled 12 h:12 h light:dark cycle with the light on at 08:00. All experiments were approved by the Animal Care and Use Committee at Chungnam National University (CNUH-019-A0081, 31 Dec 2019) and were consistent with the ethical guidelines of the National Institutes of Health. In the first experiment, we treated animals with medication before the stroke and compared the infarction size and behavioral test results. In the second experiment, we induced the first stroke on the right hemisphere of rats and treated them for seven days, and then compared the infarction size and behavioral test results.

### 4.2. Rose Bengal Injection and Photothrombosis

The Rats were randomly divided into four groups of five rats each. All experimental groups were gender-balanced. All rats received intraperitoneal (i.p.) injections of RB (Sigma, St. Louis, MO, USA) at least 30 min before photothrombosis. Anesthesia was induced with isoflurane in air and oxygen (1:1) during the surgical procedure. The anesthetized rat was placed in a stereotaxic frame, the scalp incised, and the laser was positioned in the sensory-motor areas of the right hemisphere. Pilot experiments indicated that 100 µL injection of 5 mg/mL RB (25 mg/kg) and 1 min pulse of photic stimulation produced proper focal cerebral infarction one day after the stroke was induced in P7. The body temperature was maintained at 37 °C by an automatic digital heating pad.

### 4.3. Pre-Stroke Medications

Before photothrombosis, control distilled water, ASA (Sigma, St. Louis, MO, USA), Clop (Sigma, St. Louis, MO, USA)or CoQ10 (Sigma, St. Louis, MO, USA) were administrated orally via the gastric tube once a day for three days. The dosages of drugs were based on rat weight as follows: ASA 30 mg/kg [45], Clop 10 mg/kg [46], or CoQ10 100 mg/kg [47]. The control rats were subjected to an identical procedure, except that no medication was mixed into distilled water.

### 4.4. Post-Stroke Medications

After photothrombosis, control distilled water, ASA, Clop, or CoQ10 were administrated orally at the same concentrations mentioned in Section 2.3 via a gastric tube after thrombosis once a day for seven days.

### 4.5. Infarction Volume

Rats were sacrificed at 24 h after photothrombosis, and the infarct volume was stained using the 1% triphenyltetrazolium chloride (TTC) (Sigma, St. Louis, MO, USA) staining method [48] with coronal sections every 2 mm. Using the Image J software (1.43, U.S. National Institute of Health, Bethesda, MD, USA), the entire brain volume and volume of increased intensity area were calculated.

### 4.6. Immunohistochemistry

For immunocytochemistry, 35 µm sections were incubated with a blocking buffer (5% normal serum/ 0.3% Triton X-100, Bio-Rad, Irvine, CA, USA) for 1 h. The tissue was serially sectioned to 35 µm thickness and immunostained with primary antibody [49]. The sections were incubated with primary anti-Iba-1 (1:200; catalog no. ab87117, abcam), anti-GFAP (1:200; catalog no. MAB360, milipore), and anti-NeuN (1:200; catalog no. 324307s, cell signaling) overnight. Nucleus staining was performed with DAPI. An Axiophot microscope (Leica, TCS SP8, Wetzlar Germany) was used for the analysis of double-stained sections. The immunodensities in the graphs were quantified using the ImageJ program software.

### 4.7. DHE Staining

Superoxide anion levels in the tissue were determined by using dihydroethidium (DHE; Thermo Fisher Scientific, Waltham, MA, USA), as described previously [50]. Brain sections were incubated with DHE (1 µM) at room temperature for 5 min and mounted on slides.

### 4.8. Evaluation of Neurological Deficits—Behavioral Tests

All behavior tests were performed by blinded observers before the stroke and post-stroke day 1. A neurological evaluation was performed to assess the motor changes of the animal one day before, and two and five days after photothrombosis by a person blinded to the group assignment. Neurological deficits were assessed using the hanging test. The rats were placed on a wire, and the time to fall after being suspended with both forelimbs was counted. Behavioral tests were performed immediately before and one day after photothrombosis.

### 4.9. Statistical Analysis

The data are represented as mean ± SD in the paragraphs and in the figure legends. The statistical analysis of the immunoblots was performed using the Mann–Whitney U-test. From the animal experiments, the resulting quantitative data across the groups were analyzed using analysis of variance (ANOVA). The Newman–Keuls method was used for posthoc analyses. A *p*-value of less than 0.05 was considered statistically significant. All statistical analyses were conducted using the Prism 6.0 software (GraphPad, San Diego, CA, USA).

## 5. Conclusions

In the present study, currently available medications administered both pre- and post-stroke reduced the scope of stroke lesions and improved behavioral tests when compared with vehicle-treated rats. This study shows that ASA, Clop, and CoQ10 could significantly have neuroprotective effects when administered immediately after neonatal stroke.

## Abbreviations

ASA	Aspirin
Clop	Clopidogrel
CoQ10	Coenzyme Q10
HI	Hypoxic ischemic
RB	Rose Bengal
tMCAo	Transient middle cerebral artery occlusion
i.p.	intraperitoneal
TTC	Triphenyltetrazolium chloride
ROS	Reactive oxygen species
